# Evaluating metagenomics tools for genome binning with real metagenomic datasets and CAMI datasets

**DOI:** 10.1186/s12859-020-03667-3

**Published:** 2020-07-28

**Authors:** Yi Yue, Hao Huang, Zhao Qi, Hui-Min Dou, Xin-Yi Liu, Tian-Fei Han, Yue Chen, Xiang-Jun Song, You-Hua Zhang, Jian Tu

**Affiliations:** 1 Anhui Province Key Laboratory of Veterinary Pathobiology and Disease Control, Anhui Agricultural University, Hefei, 230036 China; 2 School of Information & Computer, Anhui Agricultural University, Hefei, 230036 China; 3 School of Life Sciences, Anhui Agricultural University, Hefei, 230036 China; 4 School of Animal Science and Technology, Anhui Agricultural University, Hefei, 230036 China

**Keywords:** Metagenomics, Genome binning, Clustering, Benchmarking, Comparison

## Abstract

**Background:**

Shotgun metagenomics based on untargeted sequencing can explore the taxonomic profile and the function of unknown microorganisms in samples, and complement the shortage of amplicon sequencing. Binning assembled sequences into individual groups, which represent microbial genomes, is the key step and a major challenge in metagenomic research. Both supervised and unsupervised machine learning methods have been employed in binning. Genome binning belonging to unsupervised method clusters contigs into individual genome bins by machine learning methods without the assistance of any reference databases. So far a lot of genome binning tools have emerged. Evaluating these genome tools is of great significance to microbiological research. In this study, we evaluate 15 genome binning tools containing 12 original binning tools and 3 refining binning tools by comparing the performance of these tools on chicken gut metagenomic datasets and the first CAMI challenge datasets.

**Results:**

For chicken gut metagenomic datasets, original genome binner MetaBat, Groopm2 and Autometa performed better than other original binner, and MetaWrap combined the binning results of them generated the most high-quality genome bins. For CAMI datasets, Groopm2 achieved the highest purity (> 0.9) with good completeness (> 0.8), and reconstructed the most high-quality genome bins among original genome binners. Compared with Groopm2, MetaBat2 had similar performance with higher completeness and lower purity. Genome refining binners DASTool predicated the most high-quality genome bins among all genomes binners. Most genome binner performed well for unique strains. Nonetheless, reconstructing common strains still is a substantial challenge for all genome binner.

**Conclusions:**

In conclusion, we tested a set of currently available, state-of-the-art metagenomics hybrid binning tools and provided a guide for selecting tools for metagenomic binning by comparing range of purity, completeness, adjusted rand index, and the number of high-quality reconstructed bins. Furthermore, available information for future binning strategy were concluded.

## Background

Microorganisms are everywhere in the world and play an important role in geochemical cycles. In the past, culture-dependent microbiology is commonly used to study microbial ecology but it encountered a bottleneck as the majority of microorganisms are difficult to culture and isolate in laboratory [1]. As the advance of sequencing throughput and the decrease of sequencing cost, amplicon sequencing is one of the main strategies to research microbial communities’ taxonomic profiles for reasonable price, lower computing resource consumption. At the same time, some sophisticated bioinformatic tools such as usearch [2], mothur [3], dada2 [4] and qiime2 [5] were developed by trained bioinformaticians, making amplicon sequencing data analysis, including 16 s rRNA used for prokaryotic and internal transcribed spacer (ITS) used for fungal species, is friendly to most laboratory microbiologists who are unfamiliar with bioinformatic methods. One popular pipeline is amplicon sequencing analysis cooperates with PICRUST [6], which not only can get the species richness and abundance from environment samples but also can predicate function profiles of microbial communities. Nonetheless, amplicon sequencing has certain limitations owing to only phylogenetic marker genes or their parts are sequenced by specific primers, which can only provide species abundance information or limited microorganisms function contribution to microbial ecology. Besides, conventional primers may not be bound to some special 16 s rRNA [7] . The solution to the defects of marker gene sequencing is the whole metagenome shotgun sequencing. Shotgun metagenomics is untargeted sequencing (‘shotgun’) for all present microbial genomes (‘meta’) in samples [8]. The combined analysis of amplicon sequencing and PICRUST mentioned above is a cost-effective means of understanding microbial diversity. Nevertheless, PICRUST’s potential functional prediction of microbial communities is based on a comprehensive reference database of marker genes, which means it cannot predict species that are not in available databases and their potential functions. Shotgun metagenomics can address the loss of information about unknown species, such as obtaining draft genomes of uncultivated microbes, and supplement the low abundance species information that is hard to get in marker gene sequencing.

To date, metagenomics was applied to explore microbiologically diverse environments such as soil [9], gut [10], oceans [11], wastewater [12]. Undoubtedly, the microbial community is an important part of the ecosystem. The connection between microbial taxonomic composition and microorganisms function in the sample has always been one of the research hotspots of metagenomics [13–15]. The number of microbial cells in adults exceeds 100 trillion, which is as 10 times as the number of human somatic cells [16]. Therefore, applying metagenomics to study human microbiota affects our understanding of human health. Lately, Paul I Costea et al. [17] revisited the concept of enterotypes by re-analyzing accumulated data and discussed new enterotypes applications in ecological and medical contexts. The main purpose of shotgun metagenomics is to profile microbial community taxonomic composition, exploit unknown microorganisms, recover the part core or whole genome of special microbes and reveal how unknown microorganisms are involved in the metabolism of microbial communities in the environment [18]. For instance, metagenomic research can infer undescribed knowledge on antimicrobial resistance, virulence factors, and genes involved in enzyme synthesis, which may have important implications in public health, biotechnology, and pharmaceutical industries [19, 20].

Consequently, clustering or ‘bin’ assembled sequences into individual groups that represent microbial genomes is the key step and a major challenge in metagenomic research. Binning approach can be divided into taxonomic-dependent binning and taxonomic-independent binning, also called taxonomy binning and genome binning. Taxonomy binning is a supervised method to compare metagenomic sequences against a database of genomic sequences by making use of aligning algorithms such as blast [21], bowtie [22], bwa [23], minimap [24] or pre-computed databases (k-mers) of previously sequenced microbial genetic sequences. Nonetheless, taxonomy binning approach is limited by incomplete reference databases especially when focusing on understanding the metabiotic and functional contributions of unknown microorganisms contained in the sample. Genome binning approach is an unsupervised method to cluster contigs into individual genome bins by machine learning methods according to the feature patterns of sequences and linkage patterns between sequences without the assistance of any reference databases. Given the parameters used in cluster algorithms, genome binning approach can be divided into three types [20, 25, 26]: (i) sequence composition based; (ii) differential abundance based; (iii) hybrid methods that combine the sequence composition and differential abundance. Sequence composition-based binning strategies presume the sequence features from different genomes are distinct whereas the sequence features of a genome are similar. %G + C, nucleotides frequency [27] (k-mers frequency, typically 4 nt in length), essential single copy genes [20], are common used as sequence composition features. A basic condition for sequence composition-based methods is that the sequence length is the longer the better genome signature extracted from it. Moreover, the sequence number of low abundance species is lower, so their genome signature may not be representative and that low abundance species would be clustered into high abundance taxon [25]. Besides, discriminating closely related genomes is a significant challenge to sequence composition-based methods as closely related genomes have similar sequence features. With the current availability of advanced NGS (next generation sequencing) machines and increasing sequencing depth, microbial population coverage information is more reliable to obtain high quality microbial genome from metagenomic datasets. Differential abundance-based binning strategies presume that the sequences belonging to the same genome have parallel abundance in the same sample, and the sequences belonging to the same species have similar abundance distribution pattern across multiple samples, which can be used to separate closely related organisms. Meanwhile, the progress of metagenomic assemblers based on de bruijin graph make the improvement of the length of contigs or scaffolds and the number of predicated genes and incorporated sequences [28]. Not only can long contigs or scaffolds with less error by utilizing modern assembly tools can reduce the loss of sequence features but also make employing the co-abundance of taxon across multiple samples possible in genome binning. Combining sequence composition-based and abundance-based methods to complement each other with improved algorithm can get more accurate and completed binning results [29, 30], so that hybrid binning methods has gradually become the mainstream [31–35].

Indeed, reconstructing genomes from environmental samples is a major challenge in metagenomics, one of the reason is the lack of accurate quality evaluation reports of binning results. To make a robust inference and optimize the binning algorithm, a general standard for comparing binning results is necessary. The Critical Assessment of Metagenome Interpretation (CAMI) is a community-led initiative to help compare metagenomic tools independently and comprehensively [36, 37]. Several genome binning tools have previously been evaluated in the first CAMI [38], but newer tools and newer version of classic binning tools requires ongoing evaluation. Here, we have evaluated 15 genome binning tools comprising of 12 original binning tools and 3 refining binning tools by comparing the performance of these tools on a chicken gut dataset (4 faecal samples) and the first CAMI challenge datasets.

## Results

In this study, we evaluated 12 original genome binning tools containing GroopM [32], MetaBat [35], MaxBin [33], SolidBin [39], Vamb [40], MetaWatt [41], Binsanity [42], Autometa [43], BMC3C [44], COCACOLA [34], CONCOCT [29], MyCC [45] and 3 refining binning tools (metaWRAP refinement module [46], Binning-refiner [47], DAS Tool [48] (Table 1)). DASTool, Binning-refiner and MetaWRAP refinement module are three metagenomic refining binner combining the results of different metagenomic original binner.

**Table 1:** Summary of twelve original genome binner and three refinning genome binner

Genome binner	Parameters	Model	Version to validate	Publication	Last update	Resources
MaxBin	k-mer frequencies, coverage, single-copy genes	Expectation-maximization, bin number estimated from single-copy marker gene analysis	2.2.6	2014	2019	https://sourceforge.net/projects/maxbin
MetaBat	4-mer frequencies, coverage	Modified K-medoids algorithm	1&2.13	2015	2020	https://bitbucket.org/berkeleylab/metabat/src/master
Groopm	coverage, contig’s length, tetranucleotide frequency	Two way clustering, Hough partitioning, self-organizing map	2	2014	2017	https://github.com/timbalam/GroopM
CONCOCT	k-mer frequencies, coverage	Gaussian mixture models, bin number determined by variable Bayesian	1.0.0	2014	2019	https://github.com/BinPro/CONCOCT
MyCC	k-mer frequencies, coverage (optional), universal single-copy genes	Affinity propagation	1	2016	2017	https://sourceforge.net/projects/sb2nhri
MetaWatt	tetranucleotide frequency, coverage	Firstly clustering by empirical relationship of the average standard deviation at tetranucleotide frequency mean, then employing interpolated Markov models	3.5.3	2012	2016	https://sourceforge.net/projects/metawatt
BMC3C	frequency variation of oligonucleotides, coverage, codon usage	Ensemble k-means, construct a weigh graph and partition it by Normalized cuts [49, 50]	\	2018	2018	http://mlda.swu.edu.cn/codes.php?name = BMC3C
Binsanity	coverage, tetranucleotide frequency, percent GC content	Affinity propagation	0.2.8	2017	2020	https://github.com/edgraham/BinSanity
Autometa	sequence homology, single-copy genes, 5-mer frequency, coverage, single-copy genes	Lowest common ancestor analysis, DBSCAN algorithm, supervised decision tree classifier recruite unclustered contigs	\	2019	2020	https://bitbucket.org/jason_c_kwan/autometa/src/master
COCACOLA	k-mer frequency, coverage, co-alignment, paired-end read linkage	K-means based on L1 distance, non-negative matrix factorization with sparse regularization, hierarchical clustering	\	2017	2017	https://github.com/younglululu/COCACOLA
SolidBin-naive	single-copy mark genes, tetranucleotide frequencies, coverage, pairwise constraints	Semi-supervised spectral Normalized cut	1.1	2019	2020	https://github.com/sufforest/SolidBin
Vamb	​tetranucleotide frequencies, coverage	Variational autoencoders, iterative medoid clustering algorithm	2.0.1	2018	2020	https://github.com/RasmussenLab/vamb
DAS Tool	original binner output bin sets	Refine bins according shared contigs between two original binner results	1.1.1	2018	2019	https://github.com/cmks/DAS_Tool
MetaWrap	original binner output bin sets	Separating every pair of contigs in different bins, selecting the best bin sets according completion and contamination	1.2.2	2018	2019	https://github.com/bxlab/metaWRAP
Binning_refiner	original binner output bin sets, single-copy genes	Scoring bins based on single-copy genes and picking up high-score bins iteratively	1.4.0	2017	2019	https://github.com/songweizhi/Binning_refiner

### The binning results of real metagenomic dataset

Yanan et al. [51] generated the chicken gut metagenomic datasets from live poultry markets that were used for evaluation of above metagenomic genome binner. The data comprise more than 50,000 Mbp clean data after quality controlling and host genome removing. Then more than 110,000 contigs whose N50 was 12,243 were generated after co-assembled by metaSPAdes [52] and the contigs less than 3000 bp were dropped. Existing evaluation methods for real metagenomic binning usually examine the single-copy core genes discovered in most microbial genomes like tRNA synthetases or ribosomal proteins and their positional information to assess the completeness and contamination of recovered genomes [53, 54]. In this study, we used CheckM [53] to evaluated the completeness and contamination of reconstructed bins. To investigate the quality distribution of reconstructed genome bins, we calculated the F1-score representing the harmonic mean of completeness (recall) and purity (precision).

**Fig. 1:**
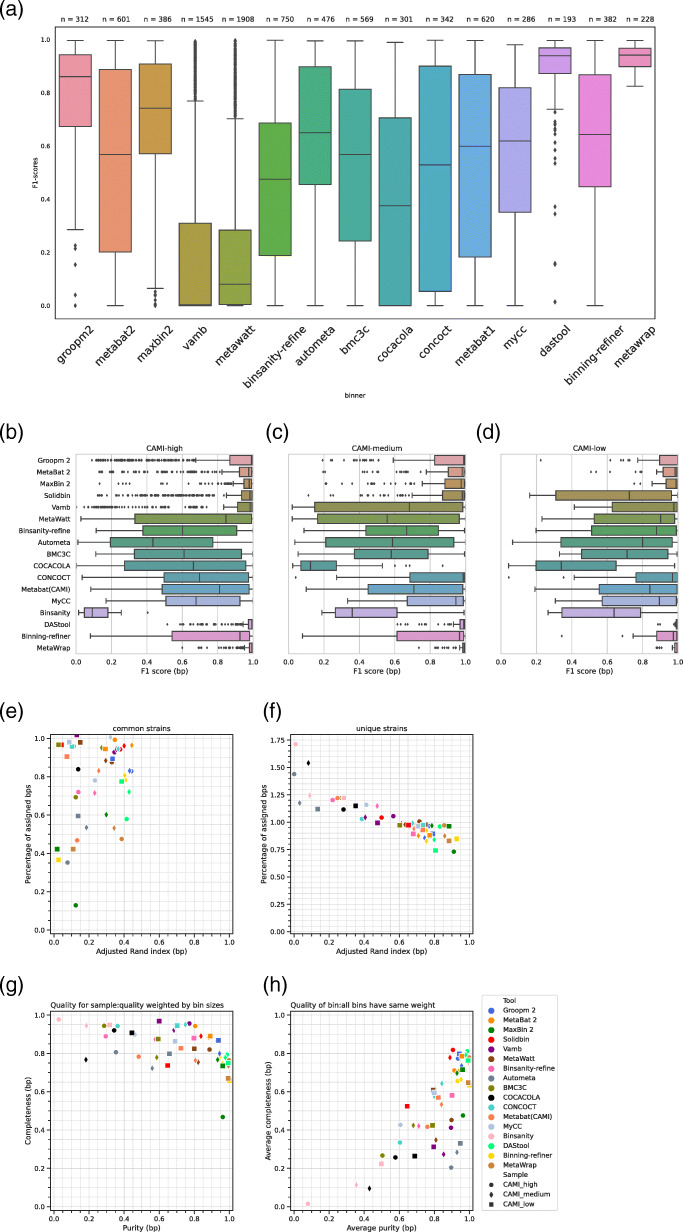
Performance of genome binning tools in chicken gut metagenomic datasets and CAMI datasets. F1-score of binning results by genome binning tools in (**a**) chicken gut metagenomic datasets and in the first CAMI challenge (**b**) high, (**c**) medium and (**d**) low-complexity datasets. (**e)** Average purity (weighted by bin sizes) and average completeness (genomes reconstructed) by genome binning tools. (**f)** Average purity (all bins have same weight) and average completeness (genomes reconstructed) by genomes binning tools. (**g)** ARI (The adjusted rand index) in connection with the segment of common strains (ANI (Average nucleotide identity) ≥ 95%) assigned by genome binning tools. (**h)** ARI in connection with the segment of common strains (ANI<95%) assigned by genome binning tools

We compared the results of above-mentioned fifteen binning predictions from the chicken gut datasets. Matawatt and Vamb predicated the greatest number of genome bins (1908 and 1545) from the real metagenomic datasets (Fig. 1), and the top 2 of average purity of recovered bins also were Matawatt and Vamb (Figure S1). Nonetheless, the average F1-score of binning results predicated by them were the lowest two (Fig. 1), which were influenced by their lower completeness (Figure S2). It indicated that Matawatt and Vamb focused on reconstructing a lot of small but pure genome bins, which may benefit the reconstruction of low-abundance microbial genome. Moreover, Vamb reconstructed 59 high-quality genome bins, reaching the intermediate level among all genome binner.

For genome original binning tools, the top 3 of the F1-socre of binning results were Groopm2, Maxbin2 and Autometa. The binners recovering the greatest number of high-quality bins were Metabat (version 1 and 2), Groopm and Autometa (87, 83 and 73 high-quality bins were recovered by Metabat, Groopm and Autometa, respectively). Generally, the more high-quality bins were combined by genome refining binner, the better the refining results were got. Hence, the bins recovered by Metabat2, Groopm and Autometa were chosen as the input of DASTool, Binning-refiner and MetaWrap (refinement module). The average F1-score of binning results from DASTool and MetaWrap was 0.89 and 0.93, exceeding all other binners, and MetaWrap achieved the greatest number of high-quality genome bins (110) from chicken gut metagenomic datasets (Table 2).

**Table 2:** The number of high-quality bins reconstructed by different binners for CAMI-high, medium, low complexity datasets and chicken gut datasets at purity greater than 0.9 and contamination less than 0.1

The number of reconstructed high-quality bins	CAMI-high datasets	Common strains of CAMI-high	Unique strains of CAMI-high	CAMI-medium datasets	Common strains of CAMI-medium	Unique strains of CAMI-medium	CAMI-low datasets	Common strains of CAMI-low	Unique strains of CAMI-low	Chicken gut metagenomic datasets
Gold standard	596	240	356	132	54	78	40	18	22	/
Groopm 2	*435	**112**	*323	**89**	32	**57**	*25	**10**	**15**	*83
MetaBat 2	*366	67	*299	77	27	50	*23	9	14	*87
MaxBin 2	236	20	216	75	21	54	*19	5	14	60
Solidbin	*403	85	*318	83	**33**	50	10	0	10	**
Vamb	364	69	295	53	12	41	13	2	11	59
MetaWatt	341	58	283	51	9	42	15	0	**15**	33
Binsanity-refine	35	2	33	16	7	9	18	4	14	27
Autometa	78	18	60	32	10	22	13	4	9	*73
BMC3C	40	0	40	22	0	22	7	0	7	64
COCACOLA	77	0	77	0	0	0	3	0	3	20
CONCOCT	71	2	69	62	9	53	15	0	15	66
Metabat (CAMI)	126	3	123	47	0	47	12	0	12	**87**
MyCC	56	3	53	45	4	41	14	0	14	20
Binsanity	0	0	0	1	0	1	2	0	2	***
DAStool	**439**	**116**	**323**	**94**	**36**	58	**29**	**14**	**15**	91
Binning-refiner	306	73	233	78	28	50	17	4	13	43
MetaWrap	427	104	**323**	91	32	**59**	22	7	**15**	**110**

*Binning results were used for the input of genome refining binner

**When Solidbin dealt with the chicken metagenomic co-assembly datasets containing more than 110 thousand contigs, it was too computing-intensive to get binning result (all the 112 threads and more than 500GB memory were used, finally Solidbin failed to return binning results)

***Binsanity provide a script Binsanity-lc comprising of binsanity and binsanity-refine to deal with the large metagenomic assemblies (> 100,000 contigs)

### The binning results on CAMI datasets

We investigated the performance of recovering genome bins of genome binners on the first CAMI challenge datasets with different complexity. For each genome binner, we used two quality weight ways to calculate average purity, one is weighted by bin size, and the other is that all bins have the same weight. The first criterion is affected by the size of recovered genome bins so that as long as the more high-abundance taxa are reconstructed, the higher purity we get. The second criterion reflect the average purity among all the predicated bins, regardless of the size of them.

For genome bins, purity (from 0 to 1) weighted by bin sizes and average completeness (from 0.4 to 1) varied considerably. For original genome binner, Groom2 had the highest purity with good completeness (> 0.9 purity, > 0.8 completeness) in three datasets, followed by MetaBat2, which had little higher completeness and lower purity (Table S5). Other two acceptable genome binner were SolidBin and MetaWatt that did excellent work in the first CAMI challenge. Besides, MaxBin2 had similar performance with Groopm2 in medium-complexity dataset. While MaxBin2 had good purity being greater than 0.9, the completeness of MaxBin2 was only 0.476 in high-complexity dataset. Remarkably, Vamb had the highest completeness with good purity (> 0.95 completeness, > 0.75 purity) in high-complexity dataset. Other programs performed well in low-complexity and medium-complexity datasets, but dealing with high-complexity dataset is a challenge to them. For three refining genome binner, DAS Tool did the best work since the purity is greater than 0.99, and the completeness varied from 0.72 to 0.96 in three datasets (Table S5). MetaWRAP also performed well as DAS Tool, while the completeness of MetaWRAP is little lower than DASTool. Compared to MetaBat2, the completeness of Binning-refinement was lower, but the purity was greater in CAMI datasets.

When focusing on low-abundance microorganisms, whose sequence composition features are more inconspicuous than high-abundance genomes in samples, investigating the average purity with the premise that all bins has same weight is a reasonable choice. As shown in Fig. 1f, genome binners such as Groopm2, MetaBat2, DASTool, MetaWRAP, SolidBin (in high-complexity and medium-complexity datasets) and MaxBin2 (in medium-complexity and low-complexity datasets) performing well as aforementioned were in the first echelon (completeness from 0.7 to 0.85, purity from 0.85 to 1). The completeness of some genome binners like Vamb and MetaWatt has declined, meaning that they were better at reconstructing high-abundance taxa, and the performance of clustering low-abundance taxa need to be improved, which we also mentioned in aforementioned evaluation to chicken gut metagenomic datasets.

To investigate how well predicated genome bins represent the reference genomes, we calculated the adjusted rand index (ARI) of recovered bins and the number of high quality bins (< 5% contaminations; > 90% completeness). For unique strains, most genome binner performed well. The percentage of assigned base pairs for all genome binner were greater than 60%, and most of them were greater than 80%. Meanwhile, the adjusted rand index for all genome binners is between 0.45 and 0.95. For original genome binner, MaxBin2 performed best with the highest ARI in high, medium and low-complexity datasets (0.884, 0.786 and 0.911). In addition, MaxBin, MetaBat2 and MetaWatt also had good performance across three CAMI datasets, while the other binning programs met the obstacle in high-complexity dataset. For common strains, the adjusted rand index of all genome binners declined substantially (< 0.4) comparing with unique strains, whose ARI were above 0.6. On the other hand, the percentage of assigned base pairs of genome binners deceased significantly as well. Among genome binners, Groopm2, MetaBat2, SolidBin, Vamb and DASTool performed relative well. The highest ARI in high-complexity dataset is 0.441 from Groopm2, in medium-complexity dataset is 0.444 from MetaBat2 and in low-complexity dataset is 0.386 from DASTool. Only Groopm2 and DASTool reconstructed more than half gold standard high-quality genome bins in medium and low complexity datasets. As aforementioned, the binning results from original binners recovering the top 3 number of high-quality genome bins were combined as the input of genome refining binners. DASTool produced maximum high-quality genome bins (439, 94 and 29) among all genome binners for three CAMI datasets (Table 2).

### Refining of original binning results

In our study, the bin sets generated by MaxBin2, MetaBat2, Groopm2 and Solidbin are used as the input of refining genome binner to obtain high quality bin sets (Table 2). DASTool, Binning-refiner and MetaWRAP (refinement module) are three published and first-class genome binning programs for refining original binning results by consolidating and improving bin sets. For instance, for CAMI high-complexity dataset, the number of high contamination (> 0.4) bins for MetaBat2, Groopm2 and Solidbin exceeded 65, after refining by DASTool and MetaWrap, the number of contaminated bins were much lower than the original binning results (Figure S3); for CAMI medium-complexity datasets, the heatmap of confusion matrices of binning results from Groopm2, MetaBat2 and Solidbin showed that even the predicated bins were generated by the first-class original genome binner, a considerable part of which is a combination of contigs from different microbial strains, that is, contaminated genome bins (Table S5a, S5b and S5c), after refining by DASTool and MetaWrap, the number of contaminated bins were greatly reduced (Table S5d and Table S5e).

## Discussion

For chicken metagenomic datasets, original genome binner MetaBat, Groopm2 and Autometa performed good than other original binners, and MetaWrap combined the binning results of them generated the most high-quality genome bins. For CAMI datasets, the latest iterative versions of classic original binning tools such as Groopm2 and MetaBat2 show the top-ranking performances, indicating their adaptability and flexibility to different complexity data sets. In contrast to MetaBat1 in the first CAMI challenge, the performance of MetaBat2 has been improved a lot, including an increase in the number of reconstructed genome bins, the purity of predicated bins, and the completeness of underlying genome. Newly published genome binning tools, such as SolidBin and Vamb, have similar performance compared with forefront genome binning tools in CAMI medium and high complexity data sets. Whether reconstructing large or small size genomes are required, Groopm2, MetaBat2 provided best performance metrics in recall, purity and the number of high-quality genome bins. DASTool, metaWRAP (refinement module) and Binning-refiner can reduce the contamination and increase the completeness of genome bin. DASTool generated the most high-quality genome bins among all genome binner for CAMI high, medium and low-complexity datasets. With regards to recover diverse strains, more than half of binning programs performed very well when dealing with unique genomes in CAMI three datasets. Nevertheless, dealing with common strains complicates all of binning tools. For example, over 90% of unique genomes with high quality were recovered by Groopm2 in high-complexity data set. Instead, less than 46% of common genomes with high-quality were recovered.

One of the deficiencies in our study is the absence of validating genome binners on diverse environmental samples. A genome binning strategy satisfying all the requirements in realistic study is unpractical. In diverse environment, the performance of the genome binners would be distinct. The second round of CAMI challenges was already been in progress and provided several multi-sample data sets from different environments to validate metagenomic tools [49].

In a recent study by Simon H. Ye et al. [50], the authors reported that only a small percentage of the first CAMI data sets were able to be classified at species or genus levels by taxonomy binning tools. When a high-resolution view on natural microbial communities are required, de novo assembly and genome binning of metagenomes are appropriate strategies. As aforementioned, reconstructing more higher resolution draft genomes, i.e. closely related strains, is one of the biggest challenges for current binning programs. Nucleotide frequency, %G + C profiles, single-copy genes and microbial population abundance information are the main features used by current state-of-the-art hybrid binning algorithms, which achieve considerable high-quality genome bins at unique strain level. To reconstruct common strains deriving from microbial communities, employing other parameters is necessary. Among the methods evaluated here, BMC3C is a pioneer in the use of codon usage features; Autometa separate contigs from metagenome into kingdom bins based on sequence homology as pretreatment before clustering, which can reduce eukaryotic contamination and increase the precision of genome bin; COCACOLA takes co-alignment and paired-end read linkage information to improve binning; SolidBin, a semi-supervised method, employed additional biological information such as dependable taxonomy assignment of some contigs to improve contig binning. Using above and other extra information would increase the computational burden and make the binning model more complex but could be a feasible way for future binning research.

## Conclusions

In conclusion, we tested a set of currently available, state-of-the-art metagenomics hybrid binning tools to evaluate their performances by applying them to chicken gut metagenomic datasets and the first CAMI high, medium and low complexity datasets. Original genome binner Groopm2, MetaBat2 and refining binner DASTool, MetaWrap achieved excellent performance across real and simulated datasets. As the spectacular technological and methodological advances, integrative omics analysis including marker gene sequencing, metagenomics, metatranscriptomics, metaproteomics, and metabolomics arises at the historic moment. Combining metagenomic assemblers and metagenomic binner into integrative omics analysis, which is the key to comprehensively understand the composition and function of microbial communities, is an irresistible trend.

## Methods

### Datasets

To address the lack of consistency in metagenomic genome binning software evaluation, CAMI provides three datasets with different complexity: (i) high-complexity datasets consisting of 5 time series samples with 596 genomes and 478 circular elements; (ii) medium-complexity datasets consisting of 4 samples in two different abundance and two different insert size; (iii) low-complexity datasets consisting of 1 sample with small insert size. In addition, gold standard assembly results and mapping results were provided by CAMI, which could be the input file of genome binning tools. Gold standard of assembly and binning can minimize chimera errors caused by assembly tools and reduce biases in evaluation of the performance of each genome binning tool.

The chicken gut metagenomic datasets (4 chicken faecal samples) were quality controlled by fastp [55] (−-cut_tail, −-length_required = 50, −-correction) to remove low quality sequences and aligned to chicken genome to remove host genome. After that, metagenomic clean reads co-assemblied with metaSPAdes [52].

### Evaluation criteria

We used AMBER [56] to calculate four representative evaluation metrics, *recall* (also known as *completeness*)*, precision* (also known as *purity*)*, F1-score* and Adjusted Rand Index (*ARI*), for evaluating the binning results. The classification of pairs of contigs fall into 4 cases: TP (Ture Positive) and FP (False Positive) represent the number of pairwise contigs belonging to the same genomes clustered into the same and different clusters, respectively. FN (False Negative) and TN (True Negative) represent the number of pairwise contigs belonging to different genomes clustered into the same and different clusters, respectively. *Recall*, *precision* and *F1-score* are calculated as:

$$ completeness= recall=\frac{TP}{TP+ FN} $$

$$ purity= precision=\frac{TP}{TP+ FP} $$

$$ contamination=1- purity $$

$$ F1=2\ast \frac{precision\ast recall}{precision+ recall} $$

Following the first CAMI [38] and AMBER [56], we calculated a truncated average precision value by removing 1% of the smallest predicted bins since their purity is much lower than that of large bins, and small and large bins contribute equally to the average precision. In order to allow assessment of the performance of recovering different abundant genomes for genome binning tools, the average purity per base pair and completeness per base pair were calculated. In addition, average precision of bins weighted by bin sizes were also calculated. Besides, underlying genomes in samples were divided on the basic of their average nucleotide identity (ANI) [57] into ‘unique strains’ (genome with ANI ≥ 95% to other genome) and ‘common strains’ (genome with ANI<95% to other genome) for assessing the effect of strain diversity to the genome binner [38]. Average precision (purity), truncated average precision, average precision per base pair, average recall (completeness) and average recall per base pair are calculated as:

$$ average\ precision=\frac{1}{{\mathrm{M}}_{\mathrm{p}}}\sum \limits_{i=1}^{M_p} precisio{n}_i $$

$$ truncated\ average\ precision=\frac{1}{{\mathrm{M}}_{\mathrm{r},\mathcal{a}}}\sum \limits_{i=1}^{m_r} precisio{n}_i $$

$$ average\ precisio{n}_{bp}=\frac{\sum_{x\in X}T{P}_x}{\sum_{x\in X}T{P}_x+F{P}_x} $$

$$ average\ recall=\frac{1}{{\mathrm{M}}_{\mathrm{r}}}\sum \limits_{i=1}^{M_r} recal{l}_i $$

$$ average\ recal{l}_{bp}=\frac{\sum_{y\in Y}T{P}_y}{\sum_{y\in Y}T{P}_y+F{N}_y} $$

where M_p_ is the number of all predicated bins, M_r_ is the number of real bins in datasets, ${\mathrm{M}}_{\mathrm{r},\mathcal{a}}$ is the number of bins passing the $\mathcal{a}$ percentile bin size threshold, X is the predicated bin sets and Y is the underlying genomes.

In addition, a *K* × *S* matrix can be constructed $\mathrm{A}={\mathcal{n}}_{\mathrm{ij}}$, ${\mathcal{n}}_{\mathrm{ij}}$ indicate the number of assignments to the $\mathcal{i}$ th bin and $\mathcal{j}$ th genome as Alneberg J et al. did [29]. Let *N* be the number of contigs from underlying genomes assigning to predicated genome bins. Adjusted rand index is calculated as:

$$ ARI=\frac{\sum_{i,j}\left(\begin{array}{c}{n}_{i,j}\\ {}2\end{array}\right)-\frac{\sum_i\left(\begin{array}{c}{n}_{i,\cdotp}\\ {}2\end{array}\right){\sum}_j\left(\begin{array}{c}{n}_{\cdotp, j}\\ {}2\end{array}\right)}{\left(\begin{array}{c}N\\ {}2\end{array}\right)}}{\frac{1}{2}\left[{\sum}_i\left(\begin{array}{c}{n}_{i,\cdotp}\\ {}2\end{array}\right)+{\sum}_j\left(\begin{array}{c}{n}_{\cdotp, j}\\ {}2\end{array}\right)\right]-\frac{\sum_i\left(\begin{array}{c}{n}_{i,\cdotp}\\ {}2\end{array}\right){\sum}_j\left(\begin{array}{c}{n}_{\cdotp, j}\\ {}2\end{array}\right)}{\left(\begin{array}{c}N\\ {}2\end{array}\right)}} $$

As the underlying genomes of the real metagenomic datasets were unknow, we evaluated the completeness and contamination of the recovered bins from original and refining binners by the lineage workflow of CheckM based on presence of marker gene per bin [53].

## Supplementary information

**Additional file 1: Table S1.** Binning results for CAMI-high datasets.

**Additional file 2: Table S2.** Binning results for CAMI-low datasets.

**Additional file 3: Table S3.** Binning results for CAMI-medium datasets.

**Additional file 4: Table S4.** Binning results for chicken gut metagenomic datasets.

**Additional file 5: Table S5.** Evaluation results on CAMI datasets.

**Additional file 6: Table S6.** Evaluation results on chicken gut metagenomic datasets.

**Additional file 7: Figure S1.** The purity of binning results generated by genome binning tools on chicken gut metagenomic datasets. **Figure S2.** The completeness of binning results generated by genome binning tools on chicken gut metagenomic datasets. **Figure S3.** The contamination of bins recovered from CAMI high-complexity datasets. DASTool, Binning-refine and MetaWrap combined the results of Groopm2, MetaBat2 and Solidbin. **Figure S4.** The contamination of bins recovered from CAMI medium-complexity datasets. DASTool, Binning-refine and MetaWrap combined the results of Groopm2, MetaBat2 and Solidbin. **Figure S5.** Heatmap of confusion matrices of (a) Groopm2, (b) MetaBat2, (c) Solidbin, (d) DASTool (e) MetaWRAP binning results from CAMI medium-complexity datasets, indicating the number of base parirs that were assigned to predicated bins (x-axis) generated by genome binner and underlying genomes (y-axis). **Figure S6.** Boxplot of completeness of binning results for CAMI (a) high, (b) medium, (c) low-complexity datasets. **Figure S7.** Boxplot of purity of binning results for CAMI (a) high, (b) medium, (c) low-complexity datasets.

## Data Availability

The high, medium and low complexity datasets for the first Critical Assessment of Metagenome Interpretation can download from CAMI official website. The Illumina metagenomics data of chicken faecal samples had been downloaded from the NCBI SRA database under the accessions of SRR7683033, SRR7683036, SRR7683044 and SRR7683043. The chicken genome GRCg6a was download from genbank under the accession of GCF_000002315.6.

## Abbreviation

CAMI: Critical Assessment of Metagenome Interpretation
