# Supplementary material for: Evaluating metagenomics tools for genome binning with real metagenomic datasets and CAMI datasets
Source: BMC Bioinformatics. 2020 Jul 28;21:334. doi: 10.1186/s12859-020-03667-3 (PMC7469296; doi:10.1186/s12859-020-03667-3)
Supplement: Supplementary file 7 — Additional file 7: Figure S1. The purity of binning results generated by genome binning tools on chicken gut metagenomic datasets. Figure S2. The completeness of binning results generated by genome binning tools on chicken gut metagenomic datasets. Figure S3. The contamination of bins recovered from CAMI high-complexity datasets. DASTool, Binning-refine and MetaWrap combined the results of Groopm2, MetaBat2 and Solidbin. Figure S4. The contamination of bins recovered from CAMI medium-complexity datasets. DASTool, Binning-refine and MetaWrap combined the results of Groopm2, MetaBat2 and Solidbin. Figure S5. Heatmap of confusion matrices of (a) Groopm2, (b) MetaBat2, (c) Solidbin, (d) DASTool (e) MetaWRAP binning results from CAMI medium-complexity datasets, indicating the number of base parirs that were assigned to predicated bins (x-axis) generated by genome binner and underlying genomes (y-axis). Figure S6. Boxplot of completeness of binning results for CAMI (a) high, (b) medium, (c) low-complexity datasets. Figure S7. Boxplot of purity of binning results for CAMI (a) high, (b) medium, (c) low-complexity datasets. [file 12859_2020_3667_MOESM7_ESM.pdf]

Evaluating metagenomics tools for genome binning with real metagenomic datasets and CAMI datasets--Figure Supplement

Figure S1. The purity of binning results generated by genome binning tools on chicken gut metagenomic datasets.

Figure S2. The completeness of binning results generated by genome binning tools on chicken gut metagenomic datasets.

Figure S3. The contamination of bins recovered from CAMI high-complexity datasets. DASTool, Binning-refine and MetaWrap combined the results of Groopm2, MetaBat 2 and Solidbin.

Figure S4. The contamination of bins recovered from CAMI medium-complexity datasets. DASTool, Binning-refine and MetaWrap combined the results of Groopm2, MetaBat 2 and Solidbin.

Figure S5. Heatmap of confusion matrices of (a) Groopm 2, (b) MetaBat2, (c) Solidbin, (d) DASTool (e) MetaWRAP binning results from CAMI medium-complexity datasets, indicating the number of base parirs that were assigned to predicated bins (x-axis) generated by genome binner and underlying genomes (y-axis).

Figure S6. Boxplot of completeness of binning results for CAMI (a) high, (b) medium, (c) low-complexity datasets.

Figure S7. Boxplot of purity of binning results for CAMI (a) high, (b) medium, (c) low-complexity datasets.

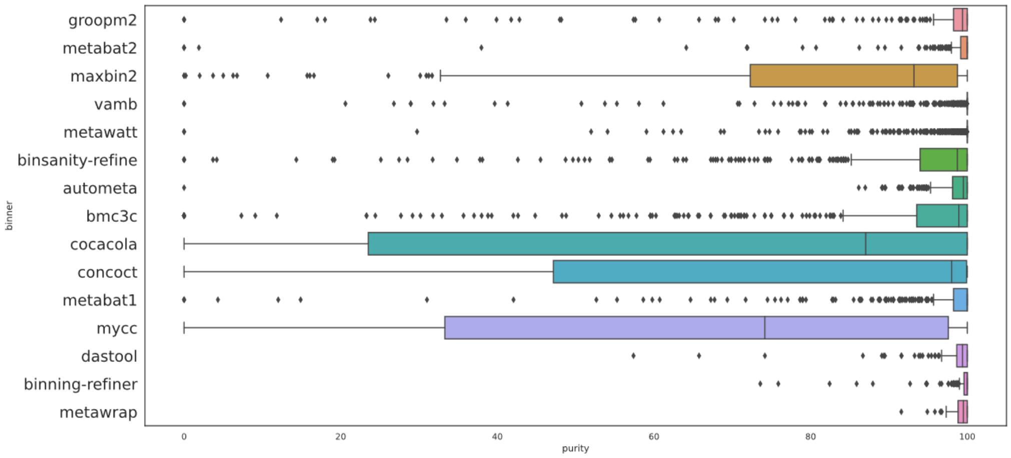

Figure S1. The purity of binning results generated by genome binning tools on chicken gut metagenomic datasets.

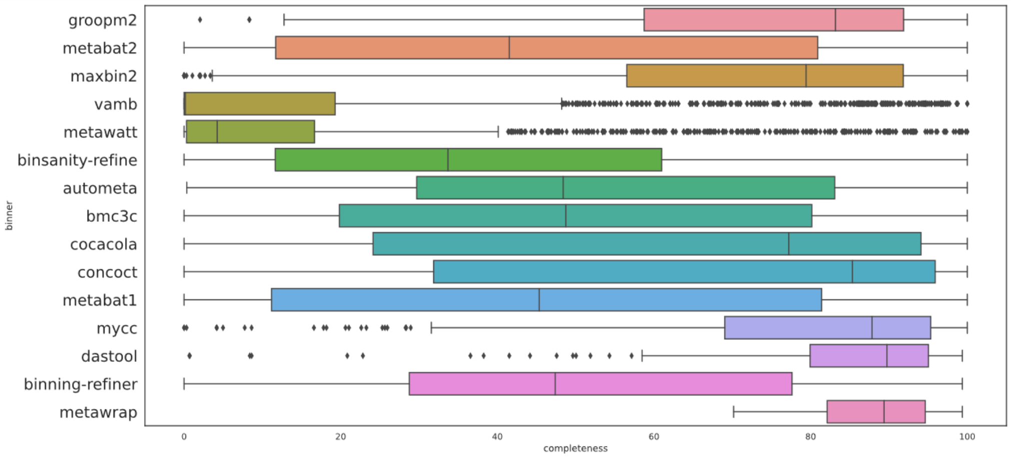

Figure S2. The completeness of binning results generated by genome binning tools on chicken gut metagenomic datasets.

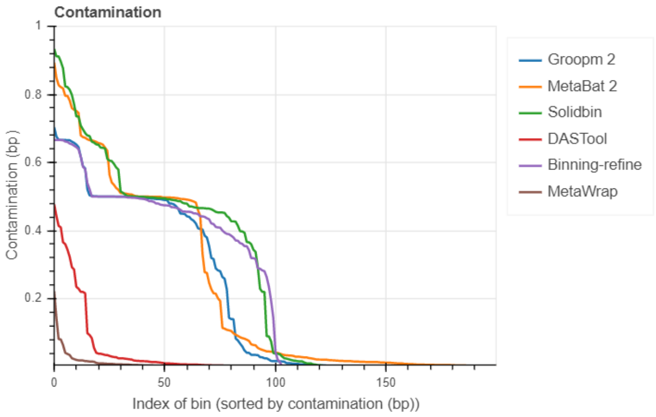

Figure S3. The contamination of bins recovered from CAMI high-complexity datasets. DASTool, Binning-refine and MetaWrap combined the results of Groopm2, MetaBat 2 and Solidbin.

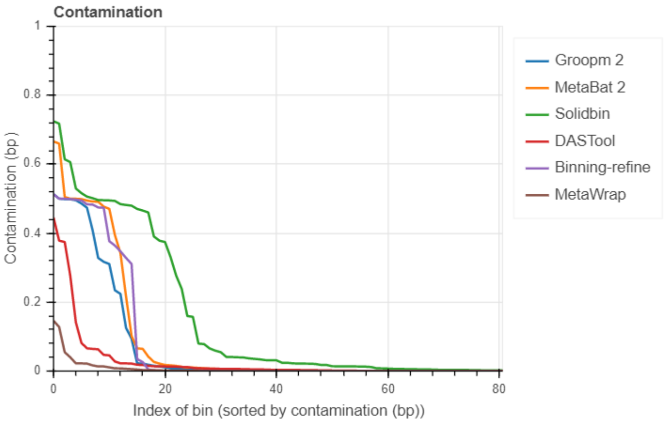

Figure S4. The contamination of bins recovered from CAMI medium-complexity datasets. DASTool, Binning-refine and MetaWrap combined the results of Groopm2, MetaBat 2 and Solidbin.

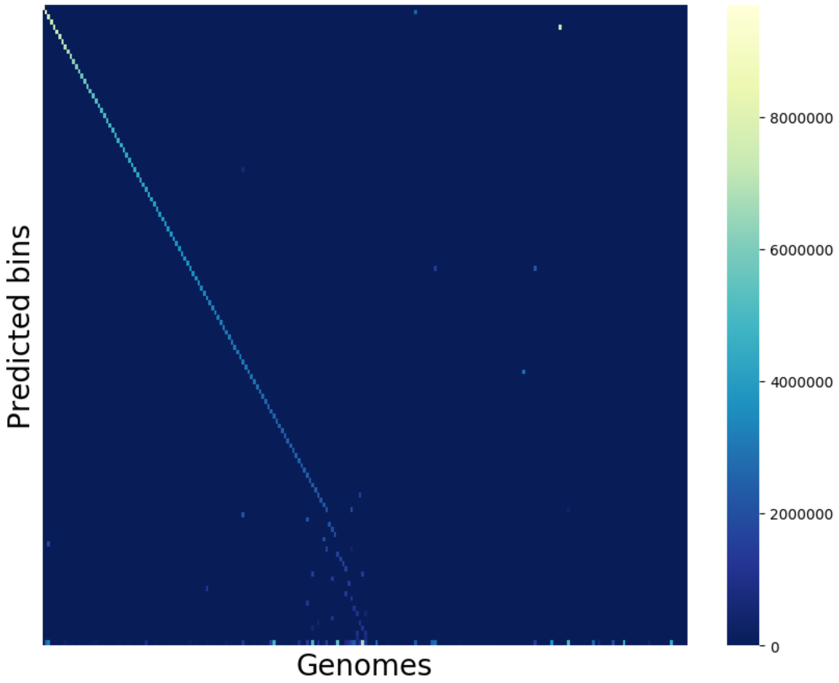

Figure S5a. Heatmap of confusion matrices of Groopm 2 binning results from CAMI medium-complexity datasets, indicating the number of base parirs that were assigned to predicated bins (x-axis) generated by genome binner and underlying genomes (y-axis).

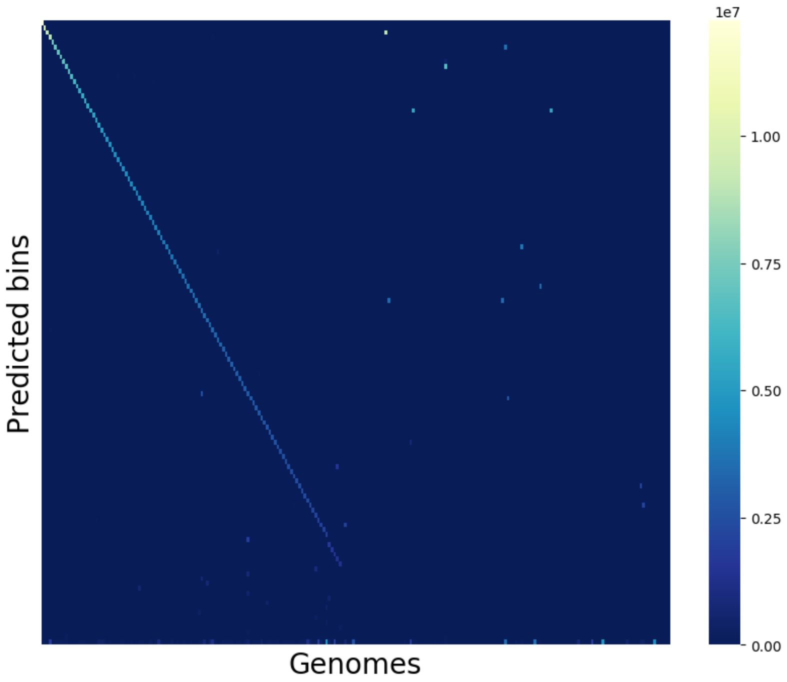

Figure S5b. Heatmap of confusion matrices of MetaBat 2 binning results from CAMI medium-complexity datasets, indicating the number of base parirs that were assigned to predicated bins (x-axis) generated by genome binner and underlying genomes (y-axis).

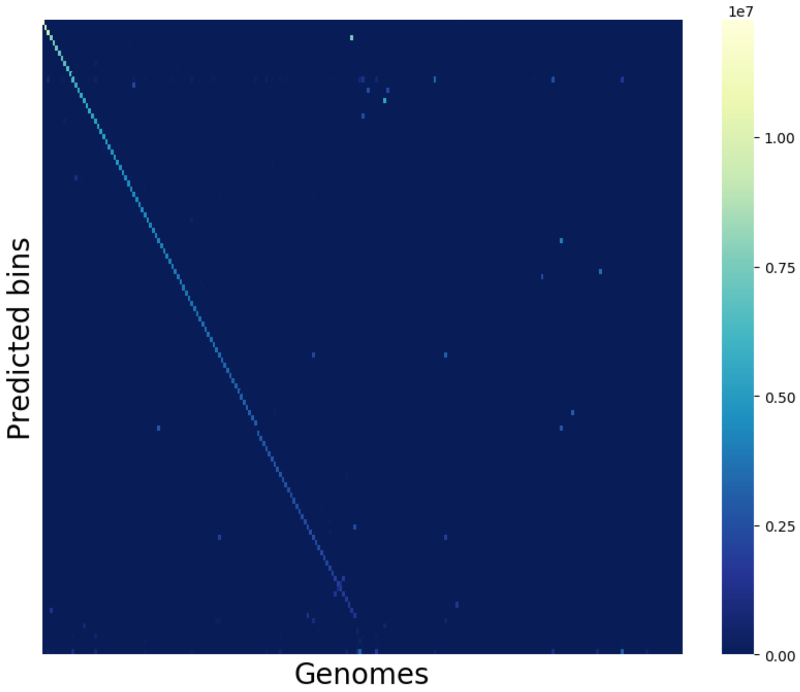

Figure S5c. Heatmap of confusion matrices of Solidbin binning results from CAMI medium-complexity datasets, indicating the number of base parirs that were assigned to predicated bins (x-axis) generated by genome binner and underlying genomes (y-axis).

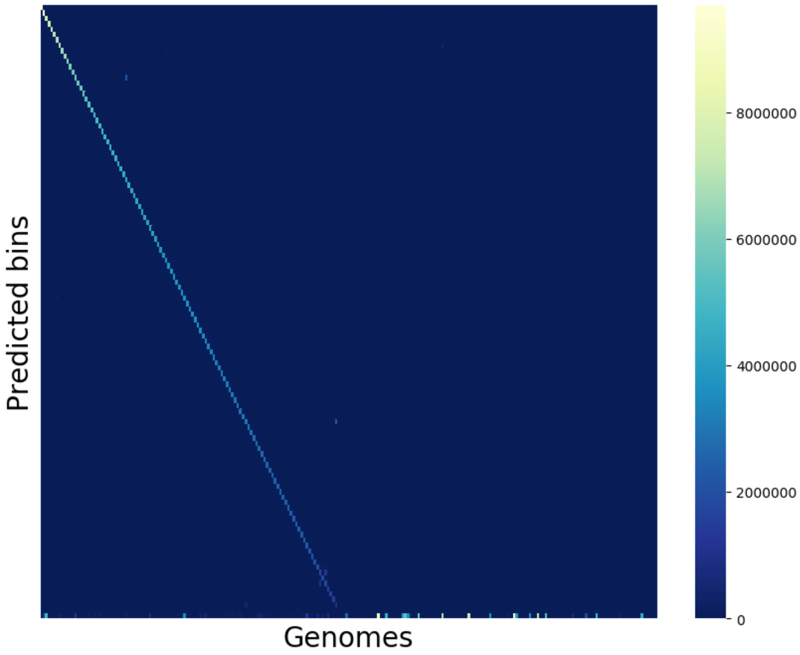

Figure S5d. Heatmap of confusion matrices of DASTool binning results from CAMI medium-complexity datasets, indicating the number of base parirs that were assigned to predicated bins (x-axis) generated by genome binner and underlying genomes (y-axis).

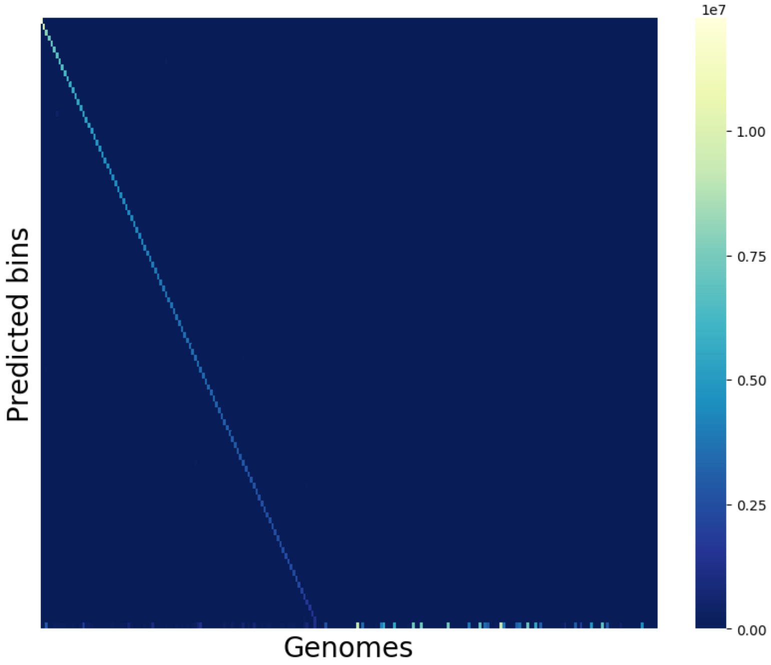

Figure S5e. Heatmap of confusion matrices of MetaWrap binning results from CAMI medium-complexity datasets, indicating the number of base parirs that were assigned to predicated bins (x-axis) generated by genome binner and underlying genomes (y-axis).

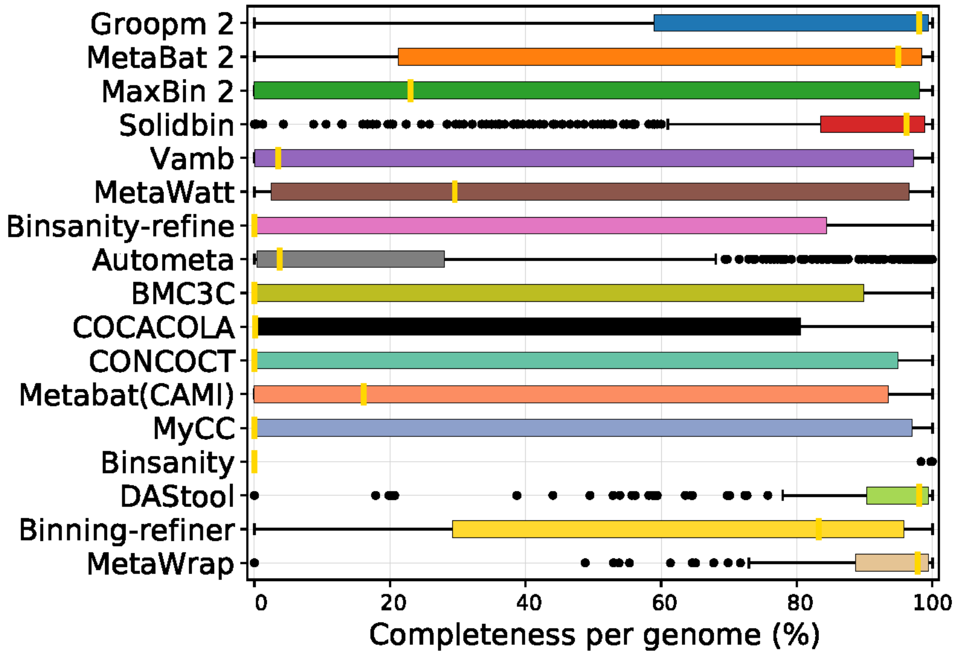

Figure S6a. Boxplot of completeness of binning results for CAMI high-complexity datasets.

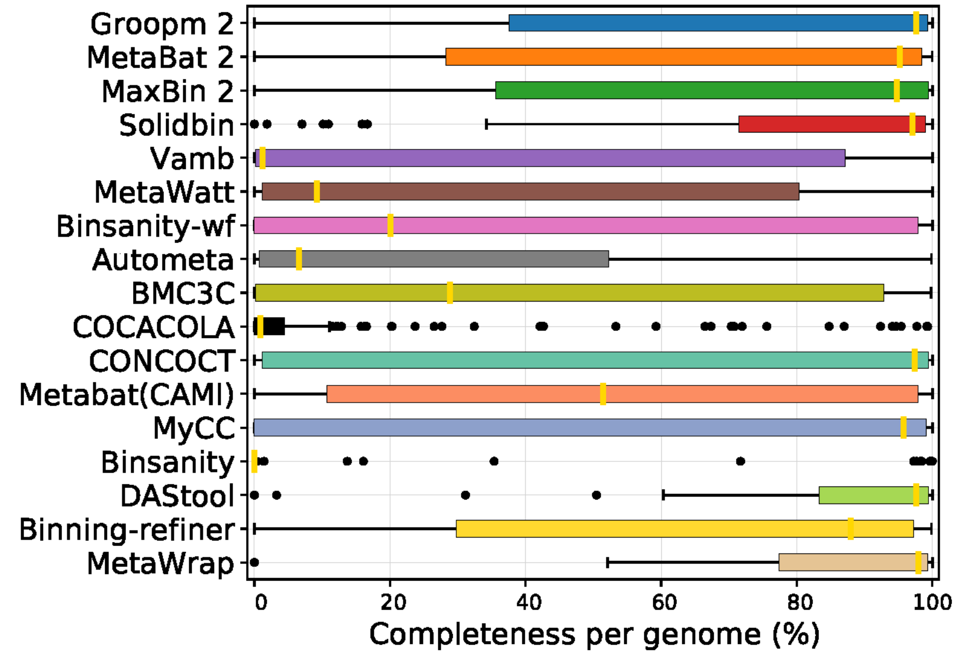

Figure S6b. Boxplot of completeness of binning results for CAMI medium-complexity datasets.

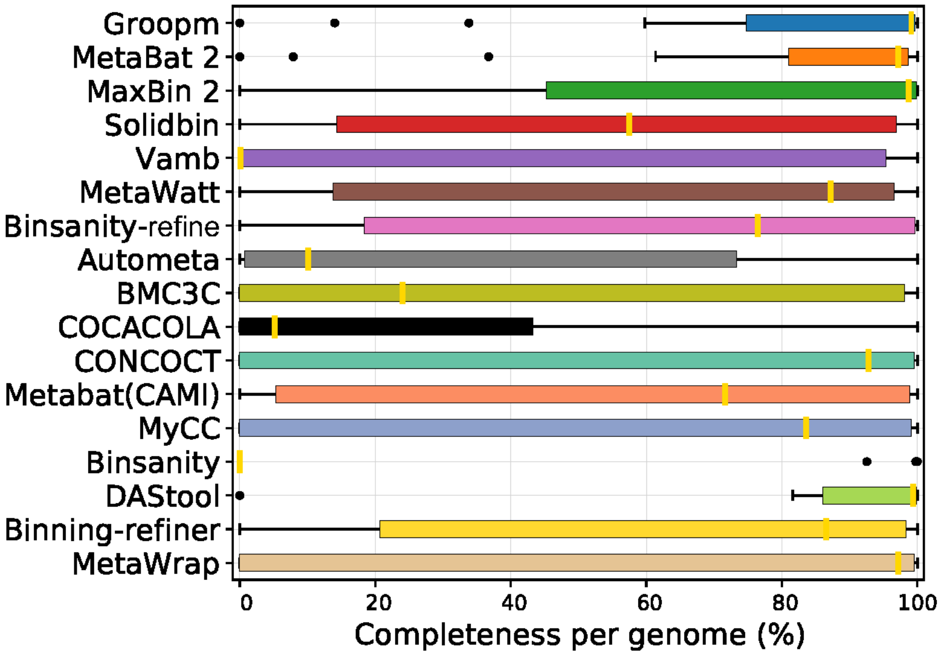

Figure S6c. Boxplot of completeness of binning results for CAMI low-complexity datasets.

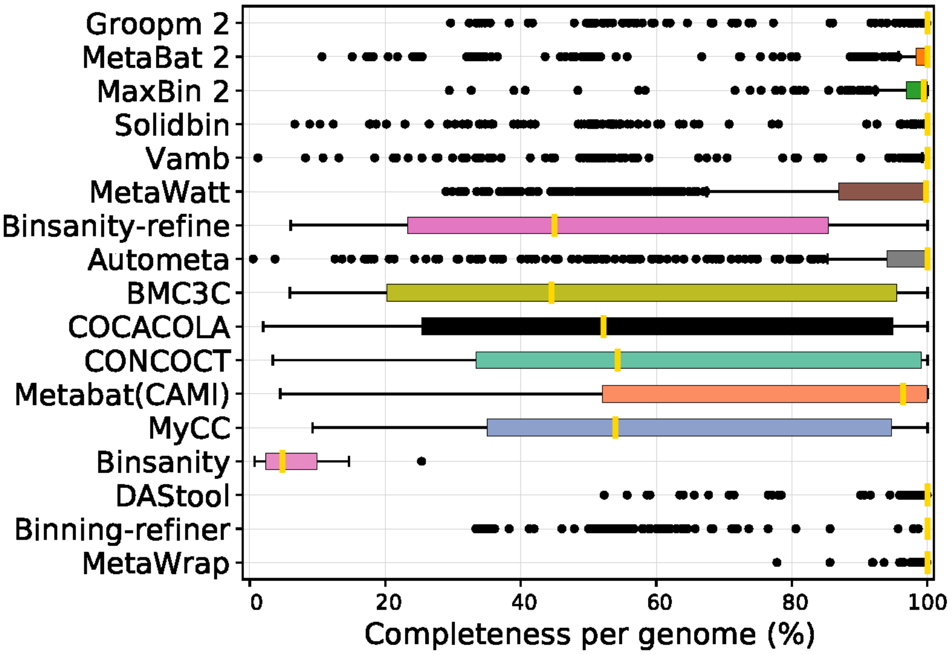

Figure S7a. Boxplot of purity of binning results for CAMI high-complexity datasets.

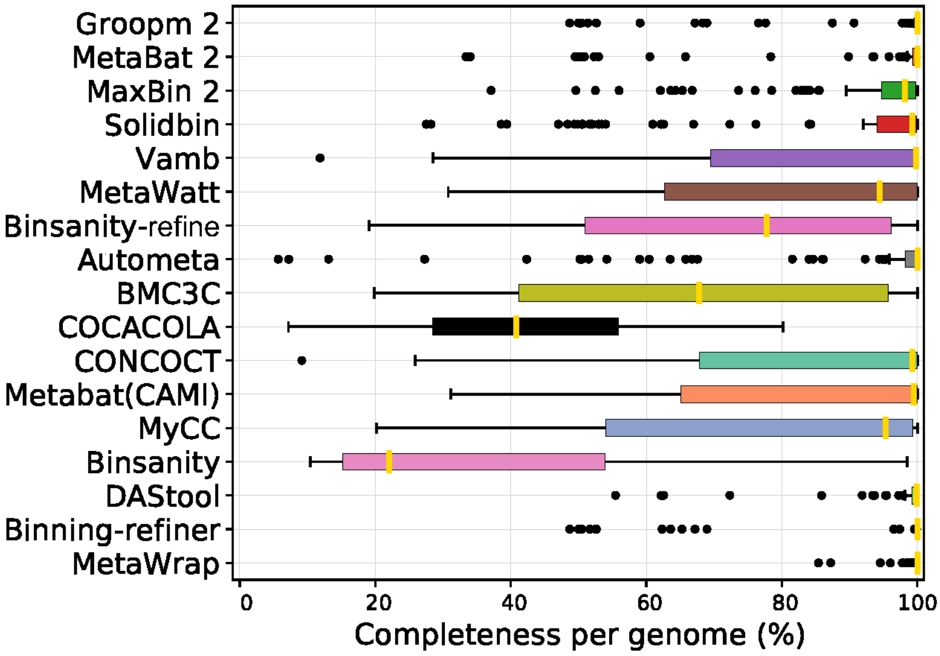

Figure S7b. Boxplot of purity of binning results for CAMI medium-complexity datasets.

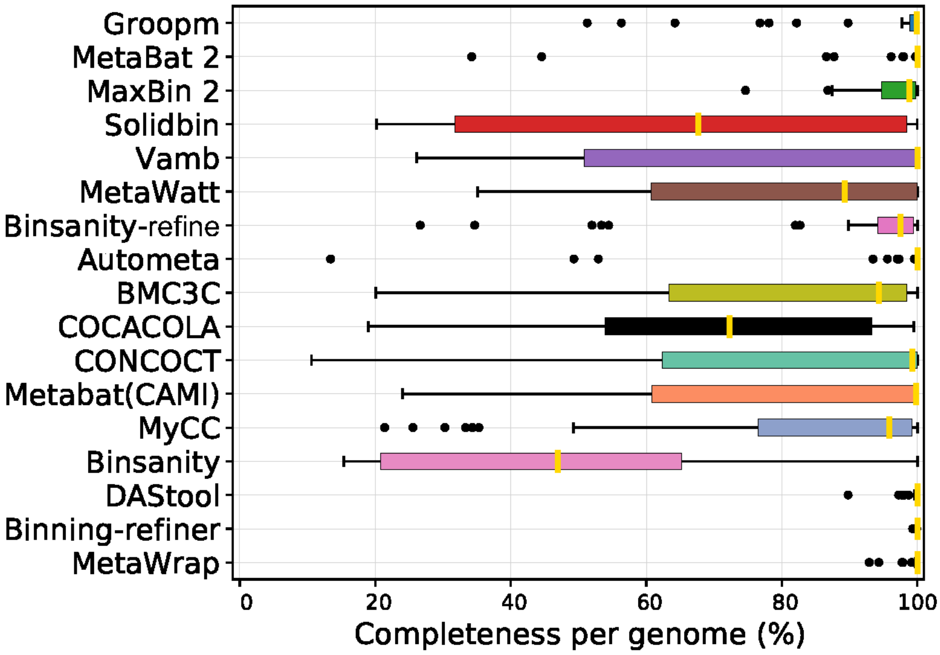

Figure S7c. Boxplot of purity of binning results for CAMI low-complexity datasets.
